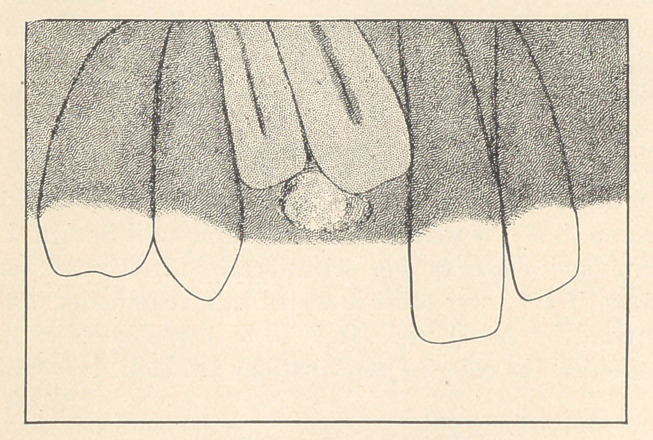# The New York Institute of Stomatology

**Published:** 1902-10

**Authors:** 

**Affiliations:** The New York Institute of Stomatology


### Reports of Society Meetings.

### THE NEW YORK INSTITUTE OF STOMATOLOGY.

A meeting of the Institute was held at the office of Dr. S. E. Davenport, No. 51 West Forty-seventh Street, New York, on Tuesday evening, April 1, 1902, the President, Dr. J. Morgan Howe, in the chair.

The minutes of the last meeting were read and approved.

COMMUNICATIONS ON THEORY AND PRACTICE.

*The President.*—Dr. Wilson has an interesting case that he desires to bring before the Institute.

*Dr. G. A. Wilson.*—The case which I have the pleasure of presenting to-night is one that has been of great interest to me during the past year and a half, and which will probably continue to interest me for some time to come. It is that of a boy fourteen and a half years of age, of excellent family, fairly robust and strong. The right upper central and lateral have not erupted, as shown in the cast. There are no inherited conditions of this sort so far as I can ascertain, the non-appearance of the teeth being caused by an obstruction which I will show you later.

Up to the time the boy came into my hands the policy had been to wait and let nature take its course. I soon decided, however, that nature needed some assistance, and after some coaxing finally prevailed upon the mother to have an X-ray photograph taken of that part of the arch, and I have the pleasure of showing you the result, the negatives and also prints from them.

From the radiograph it was plain to be seen that there was an obstruction looking something like the crown of a temporary molar embedded in the jaw directly in front and between the incisors, effectually preventing these teeth from coming down and into place. An operation was performed and the obstruction removed. It was about the size of a pea and reached well up into the process, and considerable of the bone had to be cut away to dislodge it. After its removal the ends of both incisors could be plainly seen, and seemed to be normal in shape and development. The operation was performed about four months ago, but there is apparently little if any change in the position of the teeth. Although I think I have a plan by which I can draw these belated teeth into position, I should be glad to receive suggestions regarding the case.

*Dr. E. A. Bogue.*—Is Dr. Wilson making any effort to separate the central from the cuspid?

*Dr. Wilson.*—This seems to be unnecessary, as they are apparently getting wider apart. I have been watching this point very carefully, but there seems to be no indication of their being nearer together than before the operation.

*Dr. C. O. Kimball.*—Did I understand Dr. Wilson to say that when he made the operation for removing the obstruction he came down upon the crowns of the incisors?

*Dr. Wilson.*—Yes, I could see the ends of the teeth plainly. They seemed fully developed and of normal sizes.

*Dr. C. F. Allan.*—At this age has not all the natural eruptive force of the jaw been expended, and would not any time spent in waiting now be lost? You say the teeth are moving apart. There certainly cannot be much lateral movement, because the natural movement of the teeth is forward.

*Dr. Wilson.*—The space is certainly not growing less.

*Dr. G. E. Rice.*—The discussion of the natural movement of the teeth during the process of eruption suggests a case I treated not quite two years ago,—a child whose second temporary molar had not erupted, but the gum over it was irritated. Thinking it would come down more readily, I cut away the gum over the crown of the tooth. I saw the child again recently, and the tooth was all free from gum and there was no apparent obstruction to its eruption; the tooth had advanced no farther, and the four cusps appeared at the bottom of a little well of surrounding gum-tissue.

*Dr. E. A. Bogue.*—Regarding Dr. Allan's hint, I will recall two cases, both occurring a good many years ago. One case was of a girl twelve years of age, who had no left central. By wedging apart the right central and left lateral, enough space was obtained to insert a wide artificial incisor. In a few months the left central came down into position. In the other case a right upper molar failed to come down, and as I failed to get space enough for it by my regulating apparatus, it disappeared entirely and never came down into position. Two or three years afterwards the young lady died, so there ends the history.

*Dr. H. L. Wheeler.*—I have sent to Worcester for a couple of models of some regulating that I did some years ago, thinking that it might be of interest. The case is of a woman thirty-two years of age. The centre of the arch was considerably to the left of the median line of the face. The right upper lateral was missing entirely, and the left very imperfect in form. I made an arch of two pieces of wire, using the wire of Ash & Sons for tube teeth. One I fitted around the lingual and one around the labial surfaces of the upper teeth. For about one-third the length of this wire I cut a screw-thread, so that it would come immediately in front and behind the central incisors. I made some nuts from a five-cent nickel, and after placing them on these threads resoldered the piece again, so that it made one continuous wire with a screw-thread, one-third of its length in the centre. Then by putting a German silver wire between the teeth and turning these nuts gradually I was able to bring these teeth into the proper position regarding the median line. By restoring the right lateral with an artificial crown, and making a shell with a porcelain facing for the left lateral, the effect was very gratifying. You can probably understand the case better from these models.

*Dr. T. W. Onderdonk.*—Referring to the case of Dr. Wilson, I find that the measurement from a point anterior to the right cuspid to the centre of the median line is about one-eighth of an inch different from that of the other side, and the radiograph shows that apparently a little notch occurs on the side of the central against which these unerupted teeth rest. I judge there is not space enough to allow these teeth to come down. I would advise an increase of space between these two teeth.

*Dr. J. N. Farrar.*—Though the subject is very interesting, I do not know that I can suggest anything that has not been mentioned already. There seems to be a discrepancy between the picture and the cast here shown, but I can readily understand that the cast may be imperfect. There is lack of room for two teeth, and I think the first thing to do would be to make sufficient space. In such cases I sometimes put in a little plate bearing an artificial tooth and further separate the adjacent teeth by gold fixtures on each side of the artificial tooth. Cases treated in this manner have generally proved successful. When there does not seem to be natural (physiological) force enough in the socket, some mechanism may be necessary to draw the tooth down. One good method is by getting hold of the tooth with a small wire slip-noose, and, attaching this to a plate, arranging a screw that can be turned a certain degree every day.

Sometimes the irritation from separating adjacent teeth is sufficient to start delinquent teeth down, but whether it would work in this case I cannot say. It has been claimed by somebody that the normal process of erupting teeth is an inflammatory action. The X-ray process has been of great service to dentists in this line of work, and I wish a plan would be made whereby a picture may be easily secured without risk to the patient.

*Dr. C. F. Allan.*—I would like to put on record a matter of heredity in connection with anomalies in dentition in a family of patients who have been under my professional care during all the years of my practice. One of the very first patients whom I ever attended was a young man of this family a little my senior, who came to me with an aching upper sixth-year molar. He had a particularly good set of teeth, and this aching molar was almost perfect. I remember there was one little gold filling on the morsal surface, with no other break in the integrity of the tooth, and yet it was aching.

My treatment would be a long story which I will not burden you with; it ended in the extraction of the tooth, much to my patient's relief and my own disgust.

Perhaps three or four years afterwards he came to me and wanted to know what "that meant," calling my attention to a little conical tooth partially filling the space of the extracted molar.

This supernumerary tooth, practically formless and with very little root, was easily taken out, and its presence, not thought of in the time of trouble, of course readily explained the discomfort he had had some years before.

A brother of the one I speak of had perfectly formed fourth molars posterior to the wisdom-teeth, but about as much smaller than these teeth as the wisdom-tooth is smaller than the second molar. He had one on each side in the upper jaw.

A daughter, at present about forty-five years of age, has been coming to me some thirty years or more. Her teeth have required much attention. About five years ago she came to me with a fistulous opening on the buccal aspect above a sixth-year upper molar, and I could not understand it. This molar was a live tooth, and, in fact, there was not a pulpless tooth in her head. I treated the fistula with strong escharotics, and it healed entirely, but when I saw her six months later, there was another fistulous opening on the palatal side and some tenderness to touch in the first molar and second bicuspid. With these precedent cases in view, I was quite certain of my position as to the cause and the remedy, and with the loss of the sixth-year molar and the second bicuspid the trouble ended; and when I was away in Europe the following summer a supernumerary tooth of some size came through in this space.

*Dr. E. A. Bogue.*—A lady came to me recently for whom I extracted a central incisor which I hold in my hand. The root is very rough, as the tooth was lost from pyorrhœa. A bicuspid, which I also present for examination, transplanted into the same mouth stayed there ten years and two months. The absorption that we usually expect to find within five or six years in implanted or transplanted teeth was not to be found on the end of this tooth at all. The cause of the loss of both these teeth was pyorrhœa.

To-day I saw a gentlemen for whom I had extracted an old root and implanted a central incisor that had been extracted about a year, and which at the time of implanting was much too light in color. It is now firm and has assumed the same color as the other teeth. He says he expects it to last twenty-five years.

Dr. Davenport then read his paper on "Stray Thoughts about Regulating."

(For Dr. Davenport's paper, see page 736.)

DISCUSSION.

*The President.*—Gentlemen, this is a very valuable and interesting paper of Dr. Davenport's. There are several points that I am sure will call out expressions of opinion.

*Dr. Eames.*—I wish to be enlightened in regard to the judiciousness of extracting teeth. I must confess that I have not reached that position in which I do not extract teeth for the purposes of regulating. I feel that there are cases in which it is judicious to remove some of the natural teeth, and that it can be done in some cases without malocclusion or malposition. I have had some experience with cases where the irregularity has been due to a deficient development of all of the bones of the face, and when there is this lack of development the stimulation that is given to the tissues operated upon has been extended over the entire face. When the teeth were in alignment it was out of harmony with the rest of the face and a condition that was unpleasant to see. I had one case of brothers, in one of whom I was enthusiastic to preserve the natural teeth, but in the younger brother I extracted, and to-day I believe that the condition of the last case is the better of the two. Another case was that of a young lady with a thin face, teeth very much crowded, almost in a double row. I believe it would have been a great mistake to have expanded the arch enough to have brought them in alignment, rather than to have extracted as I did. I raise these points to see light if possible.

*Dr. Farrar.*—The paper covers so much ground that I hardly know where to begin the discussion of it. Perhaps a few words regarding extraction might be apropos. There is an idea among a few of our profession, some of them college professors, that it is wrong to extract teeth in regulating. I think such teaching is wrong. There are cases where extraction would be right and cases where it would be wrong. The aim should be to consider, first, facial comeliness. The objects of the correction of irregularities are, first, to improve personal appearance; secondly, to improve mastication; thirdly, improvement of speech.

In brief, the first thing we should consider in every case is, how can we most benefit the patient by the operation. If widening the arch will leave the teeth looking like a picket fence in the mouth (all teeth), it would be wrong to widen it. The size, breadth, and contour of the face have much to do with the decision, and should be considered. I believe in extraction where it is necessary for the improvement of the face. I have seen cases presented before societies in which widening the arch has distorted the face; these were cases in which extraction should have been done. On the other hand, I have seen extraction done where it was an outrage upon the patient. Extraction of the first molar often makes impossible even a fair result at regulating.

In all cases we must, of course, consider anchorage for regulating fixtures, and no tooth is so valuable as the first molar for that purpose. When the first and second molars are both present the anchorage may be said to be very firm. If the first molar be extracted, especially the lower, we are liable to have the second molar incline forward, but there are cases, of course, where we are obliged to extract a first molar. I have a case in hand now in which I extracted an upper first molar because it was dead, badly decayed, and abscessed. I could have accomplished the regulating much easier had I extracted the second bicuspid, but I did not think it was right for the good of the patient to do so.

Now, in regard to the question of depressing the teeth in their sockets. I know it can be done, but after years of observation I have come to the conclusion that it is better to grind off interfering front lower teeth than to run the risk of disturbing nutrition to the pulp by compression. It does no harm whatever to grind the ends of such teeth if it be done very carefully. If the teeth are sensitive to the grinding, it should be done a little at a time, with intervals of several weeks between. There need be no failure by this plan.

*Dr. J. Bond Littig.*—In regard to the question of extracting for regulating, I do not feel any hesitancy if I think that by extracting I can save the labor of spreading both arches and make a good result, even if mastication is not quite as good, because I think one has to take the patient into consideration. When I extract a tooth I expect that to give the best result to the patient in regard to the regulating of that case. There are always a great many ways in which one can regulate. One can spread the arch, making it very much larger than it ought to be, or can take out one or more teeth and accomplish the end with very little difficulty and annoyance to the patient.

*Dr. Chas. O. Kimball.*—I think we are indebted to Dr. Davenport for the exceedingly careful, broad and general paper he has given us to-night. It teaches us a great many things and covers the subject so generally and thoroughly as to suggest to our minds lines of thought in many different directions. I want to speak of a case in connection with the extraction of teeth for regulating. It is just such a case as Dr. Littig speaks of. The articulation is perfect in every respect, except that there is a crowded condition of the lower incisors. I made an articulated model of the case; and I wish to emphasize what Dr. Davenport has said regarding that. I do not see how even the simplest case can be studied intelligently without having carefully articulated models, so that the inner as well as the outer cusps may be seen to know exactly what one is dealing with. I took this model to a friend of mine who I knew was opposed to the extraction of teeth and said to him, "How would you regulate this case?" He said, "Really that is one of the cases where, if I ever wanted to extract, I think I should extract." I am going to do it to-morrow morning, removing a lower central incisor. The upper arch is very perfect. The teeth are small, and any broadening of the upper arch would probably leave spaces between them.

*Dr. Gillette.*—I prefer not to say very much, except that the suggestion that Dr. Davenport has in mind in speaking against extracting is the ideal condition that we would all like to attain; but many of us see cases where it would be entirely wrong to consider extracting, provided any reasonable amount of work and expense would bring about a better result; but that reasonable amount of work and expense is just one of the points that this difference of opinion hinges upon. We all of us see cases where much expense and much work for the individual is contraindicated, and we have to compromise. In theory I agree entirely with what Dr. Davenport has said in regard to extraction. I dislike very much to extract a tooth in a case of regulating, and I do not do it if I can help it, yet I see cases where I feel forced to do it for the reasons that I have suggested.

*Dr. E. A. Bogue.*—I want to make my thanks to Dr. Davenport for his very judicious paper which is enough to set us all thinking, and to express my pleasure that so reasonable a presentation of our work can be made in so short a composition. At Dr. Farrar's mention about extraction and at the representation by one or two other gentlemen present of pretty strong notions in regard to extraction, I see a general cheer goes round, and incidentally one can easily pick out the men who do not believe in letting the natural teeth stay where they ought to be. But that cheer is not as general as it was a few years ago. There is a little light creeping in even to our heads, yours and mine too. As the old editor of the *Tribune* used to say, he would be very sorry if he could not change his mind, for that would show that he could not advance. Some of our brethren here have changed their minds. I am on record as being opposed to extraction, but I have extracted four or five firmly set teeth in the last dozen years. One of them was an uppper incisor for a young lady twenty-eight years of age, and extracted for the purpose of regulating; but I do not think extraction is very often called for, as we are such short-sighted mortals that we do not know what is coming next, and unfortunately extraction takes place almost always for young people who are in process of development.

I want to bring up one case that to me was pregnant with all sorts of lessons. I had occasion to make a model of the mouth of a married lady in Paris, who one year presented herself to me for dental operation. I was interested enough to ask her where she had been previously. She said she had been to Dr. A. L. Northrup, but previous to that always to Dr. Clowes. He had taken charge of her mouth from childhood. That model was shown to Dr. Clowes one day by Dr. Ives, whereupon Dr. Clowes declaimed against the treatment of that mouth in a manner to make one's hair stand on end. He said such a man was a swindler and a quack. I came up just then and said to him, "Oh, now, Dr. Clowes, might not this case have been treated in exact accord with your ideas and teachings in regard to dental gardening?" He said, "Never was such a thing possible; the man who did this extracting was evidently perfectly ignorant or thoroughly dishonest." I finally told him that he was the man. Unquestionably he had done it with sincere and honest motives, but he had not taken into account the changes that are inevitable with passing years. This case would illustrate many others. My two thousand models will tell the story in a way that no words of mine could approach.

Now, Dr. Farrar says that the first thing we have to do in contemplating a case for regulating is to consider comeliness. A gentleman in Chicago, who is unquestionably skilful as a man who can regulate dreadful deformities, told me that he knew nothing about occlusion. That question had never presented itself to him. He thought of nothing but appearances. I then said to him, "Do you ever stop to think what happens in later years?" "No, I have thought of appearances, and that only." I am free to confess, gentlemen, that it is well to have the line laid down sharply, and this gentleman's answer was honest. Dr. Farrar says the next thing is mastication, and then that we want to be generally beneficial to our patients. Dr. Farrar's remark in this regard is perfect, and we all say, "Amen." But perhaps we do not all say "amen" to the idea that we can have an improvement in mastication when we go to work and remove certain cogs from one of two cog-wheels which play into each other, and perhaps we do not think that President Roosevelt, with all his teeth, is so very deficient in personal appearance. To be sure he shows his teeth the moment he speaks, but I think he would be a man of less force if he had less teeth. You will remember what Dr. Hopkins truly said the other night, that strong men have generally good teeth. He instanced George Washington as the exception. I don't think he spoke of Queen Victoria, but it might be suggested that tartar had something to do with the loss of both these sets of teeth. Going back to Dr. Farrar, it seems to me that he did not lay stress enough upon the development which takes place between six and twenty-six years of age. There is a very big difference when we examine the jaws of Saint Peter when he was six years old and when he was sixty, and the guides in certain European cities claim to be able to show both.

He also spoke of Dr. Davenport widening the arch and throwing the teeth all out of gear. I have known Dr. Farrar a good many years, and too well to believe that he meant that remark to refer to all men who undertake orthodontia. While a few may throw their teeth out farther than they want to, that is not the idea that I understand Dr. Davenport wishes to convey at all, but I do understand him to say that he would, as a rule, widen and enlarge most irregular arches, and by that process he would gain the room necessary to bring the irregular teeth into their proper position, and in this he is perfectly right. Now, in illustration of what comes of a careful examination of mouths without models, I want to instance a case that came before Dr. Moffatt, and we are not any of us going to claim that Dr. Moffatt was not an expert practitioner. A son of General Keyes (you see I mention names) came into his hands, and he said to me one morning, "I have ordered out two lower molars, and the patient is coming in to-morrow morning to have them out, and as he originally was sent to you, you must take them out." When the boy came in, instead of extracting the teeth, I took an impression of the jaws, and told him that Dr. Moffatt and I would have to talk the matter over first. I went in to Dr. Moffatt holding the models in my hand and said not a word. He took hold of my wrist, and after turning the models from side to side, said, "By George, Bogue, that boy's mouth would have been ruined if we had taken those teeth out." I might tell almost the same story about one gentleman whom I respect very highly, and who is now in the room. When he saw the casts he recognized the condition. Without the casts he could not, looking as carefully as he might. A great deal of what he could see was from the posterior side of the models, alluded to by Dr. Kimball.

Dr. Farrar also says that no tooth is so valuable as the first molar for anchorage. Here I wanted to pat him on the back and say, "Bravo!" Now I would like to ask another question. "How about the first molar for the support of the jaws during the eruption of the permanent teeth and the shedding of the temporary teeth? Are they not just as valuable for that?" Then Dr. Farrar almost immediately speaks about extracting a tooth because it was dead. Now, another story. I spoke of this case in Boston, and some one spoke of a perfect articulation after extracting, and I offered five hundred dollars for the models of such a case. The last letter I got came from a lady dentist in Texas. She sent her models and I criticized them carefully, pointing out the imperfections that existed. The occlusion never can be perfect after one tooth has been taken out. The case I alluded to is that of a young lady fifteen years of age, the daughter of the Russian foreign minister. When she came to me the four first molars were all in such shape that ordinarily, as dentistry goes, they would have required extracting. Of the two upper ones, one had an exposed pulp and the other was so badly decayed that the pulp was nearly exposed. The left lower molar was so badly decayed that the roots were separated and an abscess had been going on for some time, and the right lower molar had dead pulp and an abscess. I took my models, as Dr. Davenport advises, and carefully considered the case. I said, "If I extract these teeth now, bad as they are, the permanent teeth behind them will come forward, the occlusion will be lost, and she will never be able to masticate properly. I will run the risk of doing what I believe is the right thing to do." I treated the exposed pulps and healed the abscesses, and the roots were filled. I put screws into the roots of the lower left molar where the roots were separated, covered the exposed gum with gutta-percha, put a ring around it, and filled the whole thing with amalgam. The young lady came to me three years and then married and went to a distant part of Russia, but I have heard from her every year since. She has gained in health, and has a family. Her husband is the governor of the province where they live. That was twelve or thirteen years ago, and she has all those teeth yet, and during all this time has had good masticating surfaces. If she now loses all of these teeth, I feel that I have done her a great service, more than I could have done her by any extraction possible.

I hope Dr. Farrar will forgive me for constantly alluding to him. He is so very interesting that I cannot help it. He made the remark that if we extract the first molar, the second molar is liable to move forward. Then I was ashamed of him. Dr. Farrar, a man who teaches us all, to say it is *liable.* It is absolutely *sure* to throw the second molar forward, and the other results due to the throwing of that molar forward are great or little according to circumstances. He also alluded to the shortening of teeth by grinding the ends off. I may say that I have shortened a good many teeth by pressure and I have yet to see the second tooth that has died during the process of regulation. Once in my life I

found an upper central incisor dead after I had regulated a set of teeth, from what cause I do not know. I would heartily commend this suggestion of Dr. Davenport. A reason for extracting which is often the rule (I hope not by members of this Institute) was given by a gentleman practising our calling on the other side of the water, to whom I had once sent a patient with a regulating fixture in the mouth. He said to me, "Why do you put such a fixture as that on that girl's teeth? Why don't you extract the four first molars and have done with it? If you extract you get your fee, and they don't know the difference and you are better off."

*Dr. Farrar.*—It is certainly evident from the remarks of Dr. Bogue that I either made a mistake in forming my language or he made a mistake in hearing it. I am not here to criticise adversely any member's methods. What I said I will scientifically adhere to.

So far as the essayist's plans go they are practicable, even excellent. So far as the "Coffin plate" is concerned, if properly made it will do the work, and I can recommend it to a certain extent, but I do not regard the mechanism equal to some that have been devised more recently.

The remark has been made that an inventor gets into a groove. This is a new idea to me. I had always thought that it was the man who has little or no inventive ability, but who follows in the footsteps of another, who gets into a groove. Of course, the inventor who has but one idea and can see nothing else may be, and generally is, a "groove man."

So far as Dr. Bogue's criticisms are concerned, I do not mind them at all. Nothing would please me more than to reply to questions by the hour, for I am full of this subject.

Concerning permanent molars, I do not extract them unless they are dead and badly decayed. In the case I referred to this was so. But it is a wrong idea that proper antagonism cannot be made after a tooth has been extracted. As much harm can be done to antagonism by improper widening of the arch as by improper extracting.

*Dr. C. F. Eames.*—I have been much interested in the valuable suggestions made by the essayist, and I do not regret having made the trip from Boston for the sole purpose of hearing his paper.

In regard to the removal of teeth for purposes of regulating, I am not sure that I understand exactly where Dr. Bogue would draw the line. I must say that I have had cases which seemed to me to call for extraction, but these are exceptions to my general rule of practice. In these cases there was deficient development of all the bones of the face, especially in the lateral direction; what might be called a very thin face.

I have seen deformity result from widening the arch in such cases, that is to say, the teeth are unduly prominent and out of proportion with the rest of the face, which has not been affected to any appreciable extent by the operations on the dental arch.

I have, on the other hand, obtained very good results in such cases by extracting the four second bicuspids and then proceeding to regulate.

*Dr. Davenport.*—This question of extraction I do not intend to make at all prominent in the paper and I am in a sense disappointed that it should have been made so much of in the discussion. While I do believe in expanding arches, I do not think patients are discharged from my office, after the completion of the regulating, with arches that are too prominent for the comliness that, as Dr. Farrar says, is so essential. If expanded arches would not agree with the proper contour and expression of the face, then of course some other method must be taken, but I think I put enough emphasis upon that point when I spoke of the study of the casts and the face together, and the necessity of making our work conform with the needed expression. I do not take the extreme position that teeth should never be extracted, and I am perfectly willing that Dr. Littig or Dr. Farrar, or any other man with judgment, should extract teeth when they believe it necessary, as I feel sure that they would do no injury by such decision, but all men, and particularly the younger men who are forming their ideas and habits, have not the experience and judgment of those I have named and therefore it is well that a word of caution should be spoken. I can remember twenty years ago when the extraction of the sixth year molars was the panacea for all cases that needed regulation, and casts were seldom taken except where an appliance was to be made. A hasty glance at the mouth and, "Oh, extract the sixth year molars." I think we ought to be very careful in advising the extraction of teeth, and only in extreme cases will it be found necessary.

Adjourned.

FRED. L. BOGUE, M.D., D.D.S.,
*Editor The New York Institute of Stomatology.*

## Figures and Tables

**Figure f1:**